# Supplementary material for: An organic artificial cardiomyocyte
Source: Nat Commun. 2026 May 6;17:4181. doi: 10.1038/s41467-026-72584-5 (PMC13153225; doi:10.1038/s41467-026-72584-5)
Supplement: Supplementary file 1 — Supplementary Information [file 41467_2026_72584_MOESM1_ESM.pdf]

Supplementary Information

An Organic Artificial Cardiomyocyte

Dace Gao *et al.*

This PDF file includes

Supplementary Figures 1-25

Supplementary Tables 1-2

Supplementary Notes 1-4

Supplementary References 1-8

Supplementary Figures

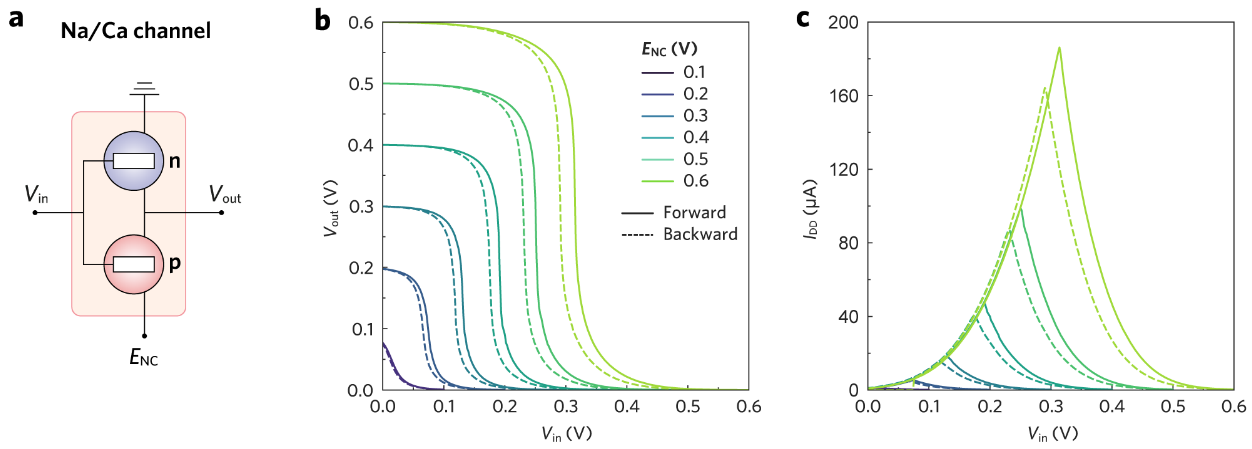

Supplementary Fig. 1 | Steady-state characterization of the Na/Ca channel, including its **(a)** equivalent circuit, **(b)** VTC, and **(c)** CTC.

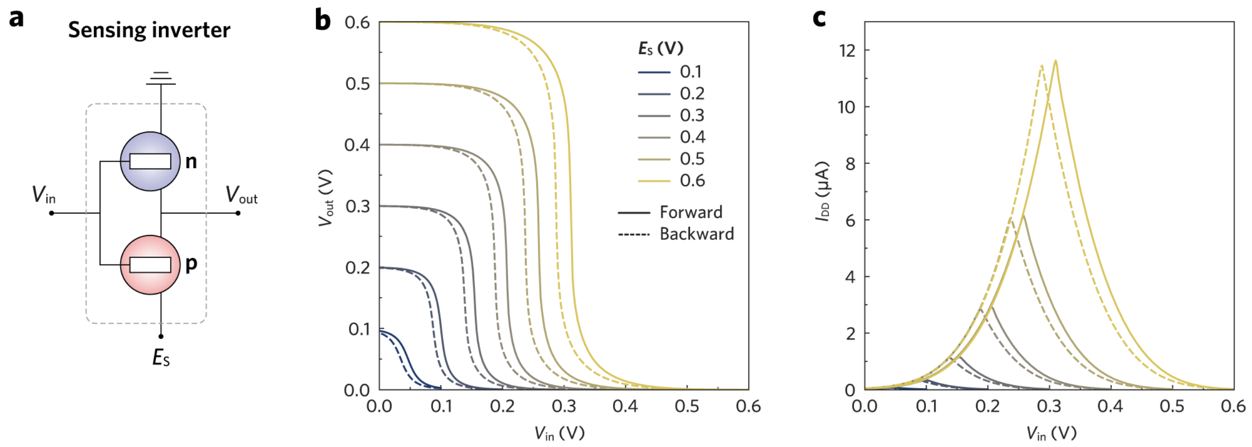

Supplementary Fig. 2 | Steady-state characterization of the sensing inverter (S-inverter), including its **(a)** equivalent circuit, **(b)** VTC, and **(c)** CTC. This thresholding inverter is denoted as a "sensing" block because of its functional analogy to the voltage-sensing domain in biological, voltage-gated ion channels. Such a channel comprises four homologous polypeptide domains, with each domain comprising 6 membrane-spanning alpha helices (S1-S6). The S4 segment is positively charged and is sensitive to the changes in transmembrane potential. At the threshold potential, the movement of the S4 voltage sensor triggers a conformational change of the ion channel's conducting pathway, regulating the opening/closing of the channel. Similarly, the S-inverter in OECM detects the changes in V_{mem} (input) and regulates V_S (output) through its VTC. The forward/backward swings in V_S triggers a conductance change in the Na/Ca channel, regulating the opening/closing of the charging pathway.

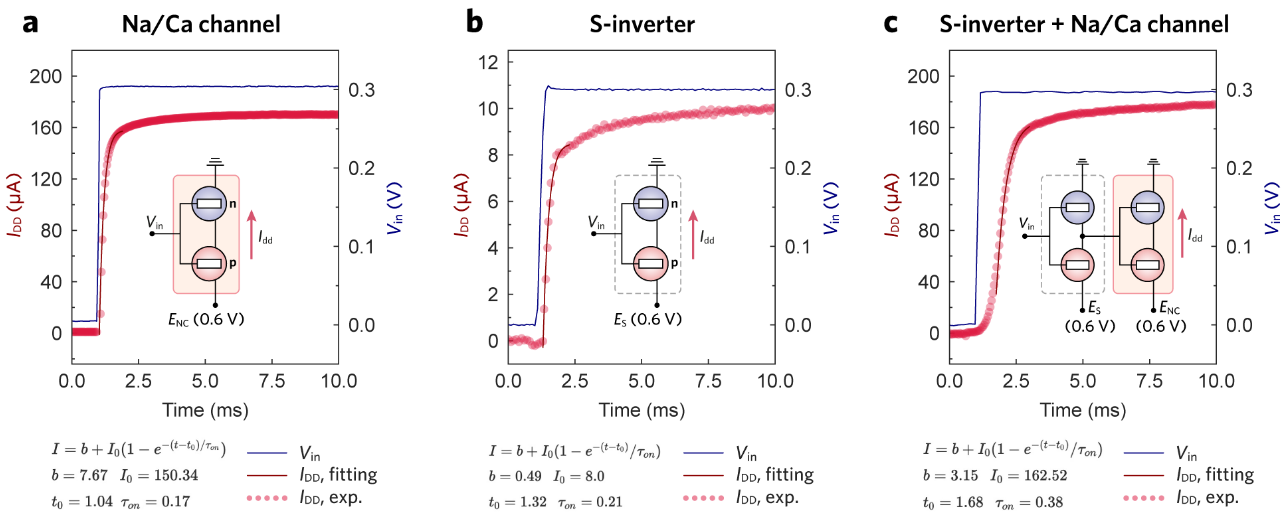

Supplementary Fig. 3 | Transient characterization and analysis of (a) the Na/Ca channel inverter, **(b)** the S-inverter, and **(c)** the cascade of these two inverters. The current rise in response to a square-pulse input voltage was recorded and fitted to an exponential rise function. The characteristic rise time (τ_{on}) for the Na/Ca channel, the S-inverter, and the cascaded inverters are 0.17 ms, 0.21 ms, and 0.38 ms, respectively. The results suggest that the Na/Ca channel can supply a charging current immediately when the S-inverter detects a threshold voltage.

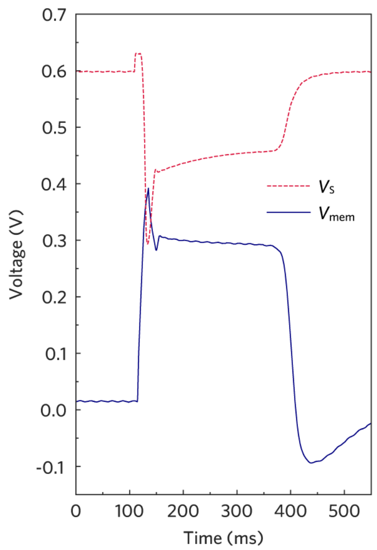

Supplementary Fig. 4 | Recording of V_{mem} and V_S over a complete OECM AP. Side-by-side plots of V_{mem} and V_S reveal their mirrored behavior during OECM operation. V_S is governed by V_{mem}, since they correspond to the output and input of the S-inverter, respectively, while V_S in turn influences V_{mem} by controlling the charging current through the Na/Ca channel.

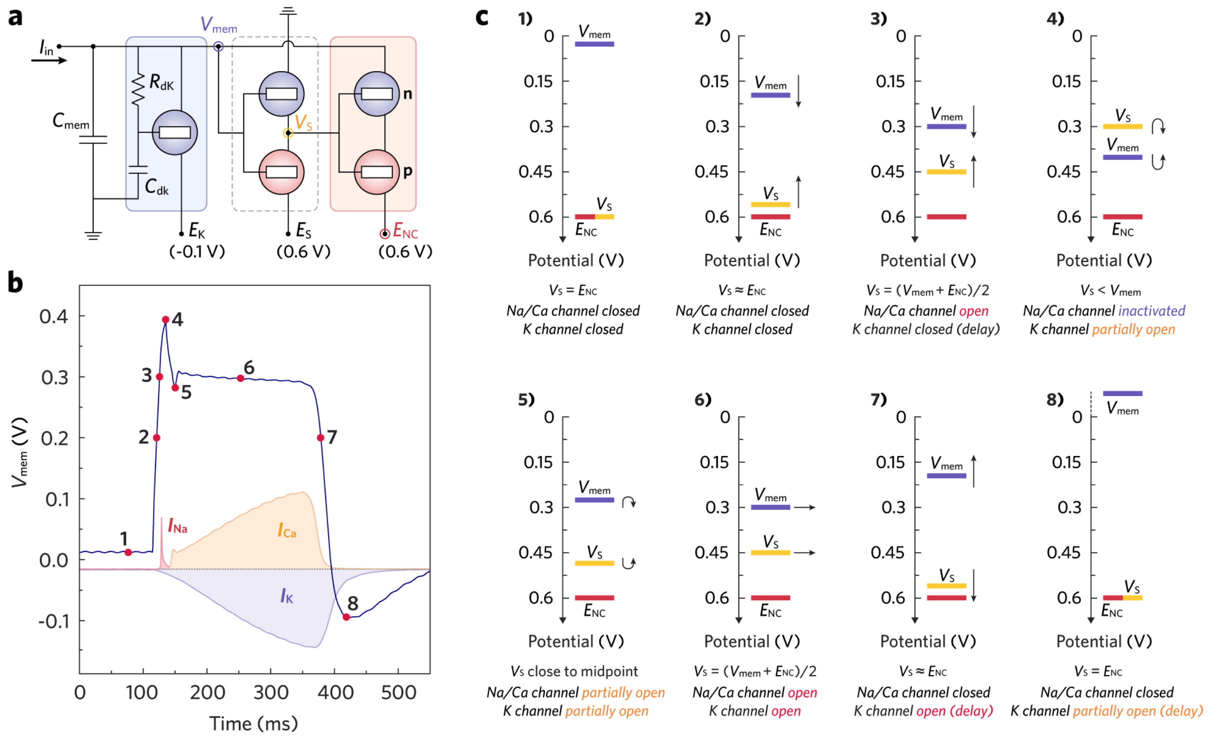

Supplementary Fig. 5 | Step-by-step analysis of an OECM's AP phases. (a) Equivalent circuit of OECM. **(b)** Recording of an OECM AP with the signature states labelled in numerical sequence. **(c)** Schematic diagrams visualizing the position of at the signature timings as labelled. The relative potential of V_S, V_{mem}, and E_{NC} determines the steady-state charging strength of the Na/Ca channel, while the relative potential of V_{mem} and E_K determines the steady-state discharging strength of the K channel. **1-2)** The I_{in} pulse charges C_{mem} and elevates V_{mem}, which squeezes the effective power rail ($E_{NC} – V_{mem}$) of the Na/Ca channel. **3)** At $V_{mem} \approx 0.3$ V, the S-inverter switches and pulls V_S down from the Na/Ca channel's upper supply voltage (~0.6 V). At the moment when $V_{mem} \approx 0.3$ V, $V_S \approx 0.45$ V, and $E_{NC} = 0.6$ V, V_S meets the midpoint of the Na/Ca channel's power rail ($V_S = (V_{mem} + E_{NC})/2$) and thus fully activates the charging current. The transient I_{Na} pulse lasts ~ 15 ms and further depolarized V_{mem}, whilst the discharging K channel remains closed due to the delay. **4)** Once V_{mem} surpasses V_S, V_S falls completely outside of the Na/Ca channel's power rail, keeping the channel inactivated. **5)** However, V_{mem} could only stay above V_S for a very short period because the K channel will gradually open and discharge C_{mem}, bringing V_{mem} slightly down below 0.3 V. **6)** At this stage, both the Na/Ca and K channels are open with paired charging and discharging strength. With ~0 net current integrated at C_{mem}, V_{mem} stabilizes at ~ 0.3 V and yields a plateau that lasts for hundreds of milliseconds. **7)** By the end of the V_{mem} plateau, the slight deviation of V_{mem} from ~0.3 V makes I_{Ca} descends rapidly, whereas I_K responses to V_{mem} change much slower. Consequently, I_K excels I_{Ca} and thus initializes repolarization. **8)** The Na/Ca channel shuts completely, whereas the K channel remains active and further discharges C_{mem}, introducing hyperpolarization. Finally, the K channel shuts and V_{mem} returns to its resting potential.

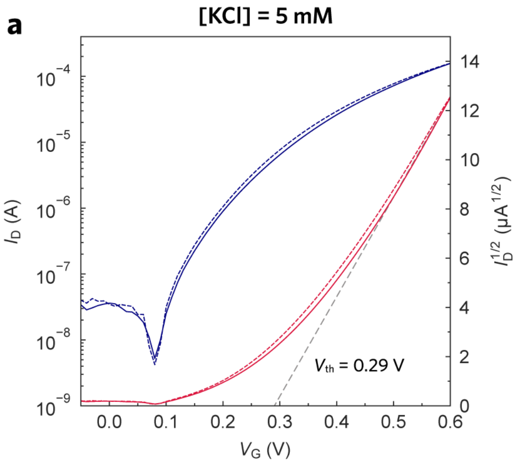

Supplementary Fig. 6 | Evaluation of the K channel OECT's threshold voltage with different electrolyte concentrations. (a) [KCl] = 5 mM, **(b)** [KCl] = 100 mM. The linear region of $I_D^{1/2}$ was extrapolated, and V_{th} was obtained as the intercept on x-axis.

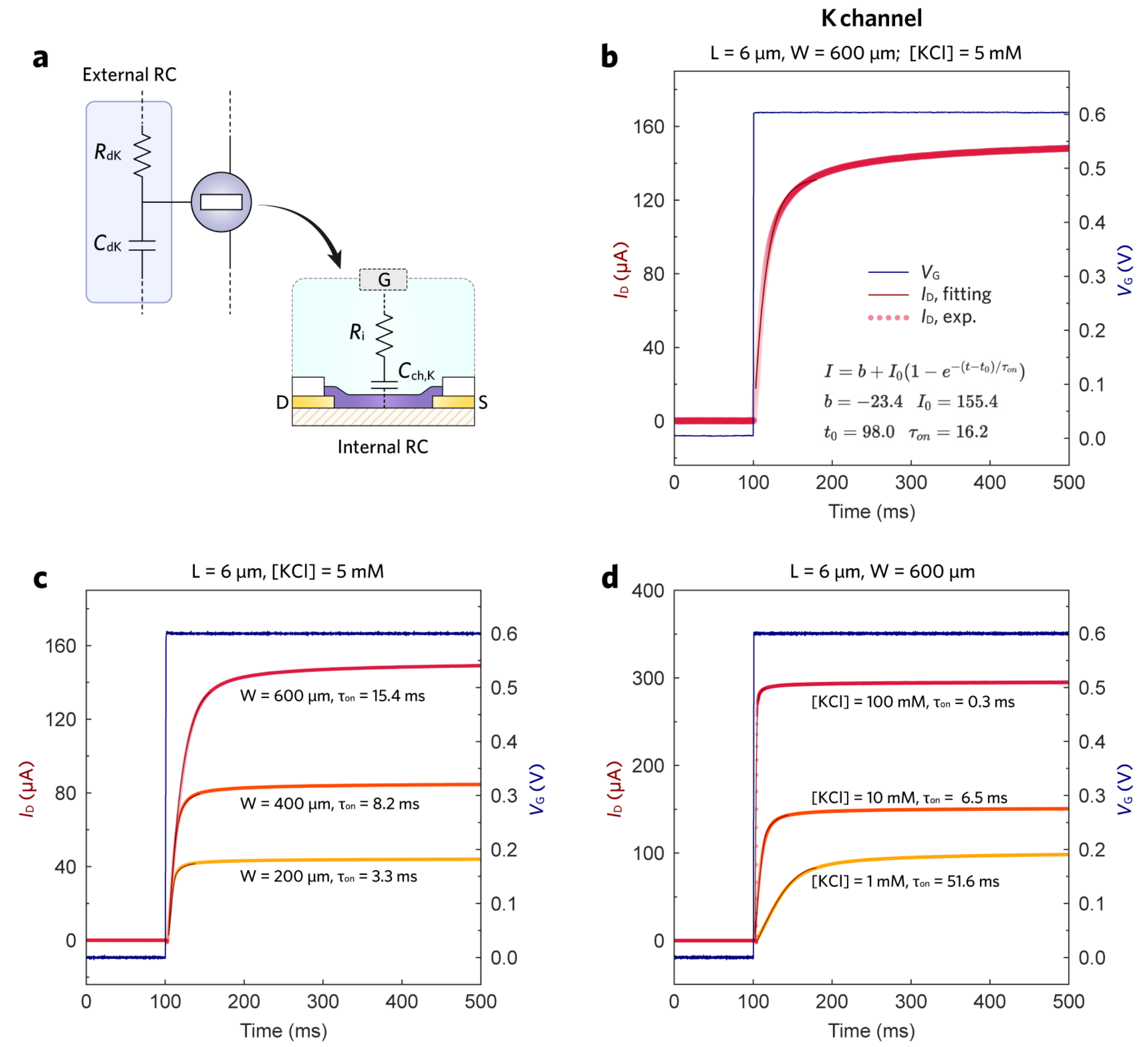

Supplementary Fig. 7 | Transient characterization and analysis of the K channel. (a) Schematic circuit and illustration that depict the external and internal RC components assigned to the K OECT. **(b)** Transient response of the K OECT employed in the OECM. The test was performed without external RC components, revealing the intrinsic RC delay. The K OECT's characteristic rise time (τ_{on} = 16.2 ms) is ~95 times larger than that of the Na/Ca channel. **(c)** Transient characteristics of the BBL OECT with different channel width, suggesting slower OECT response with larger $C_{ch,K}$. **(d)** Transient characteristics of the BBL OECT with different ionic strength, suggesting slower OECT response with higher R_i.

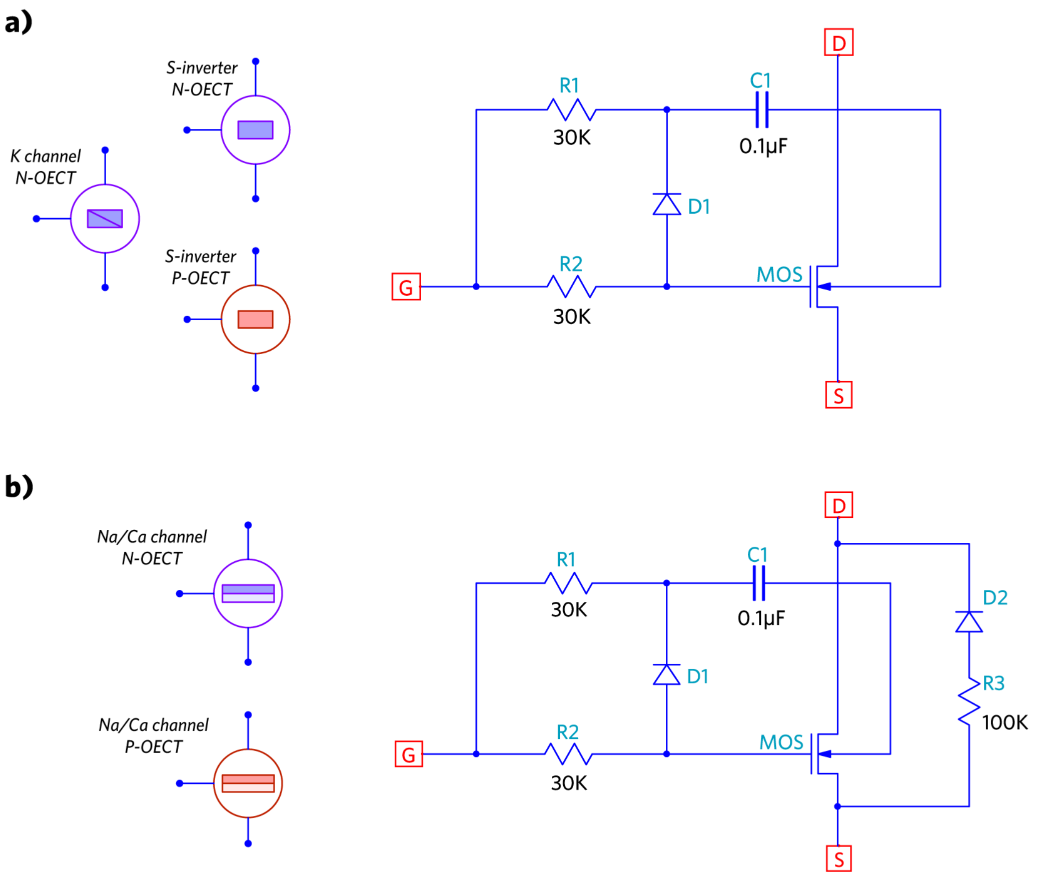

Supplementary Fig. 8 | Symbols (left) and equivalent circuits (right) of the OECT models created in SPICE. (a) Planar OECTs, including the n-type OECT in K channel, and n-type and p-type OECTs in the S-inverter. **(b)** Stair-type vertical OECTs, including the n-type and p-type OECTs in the Na/Ca channel.

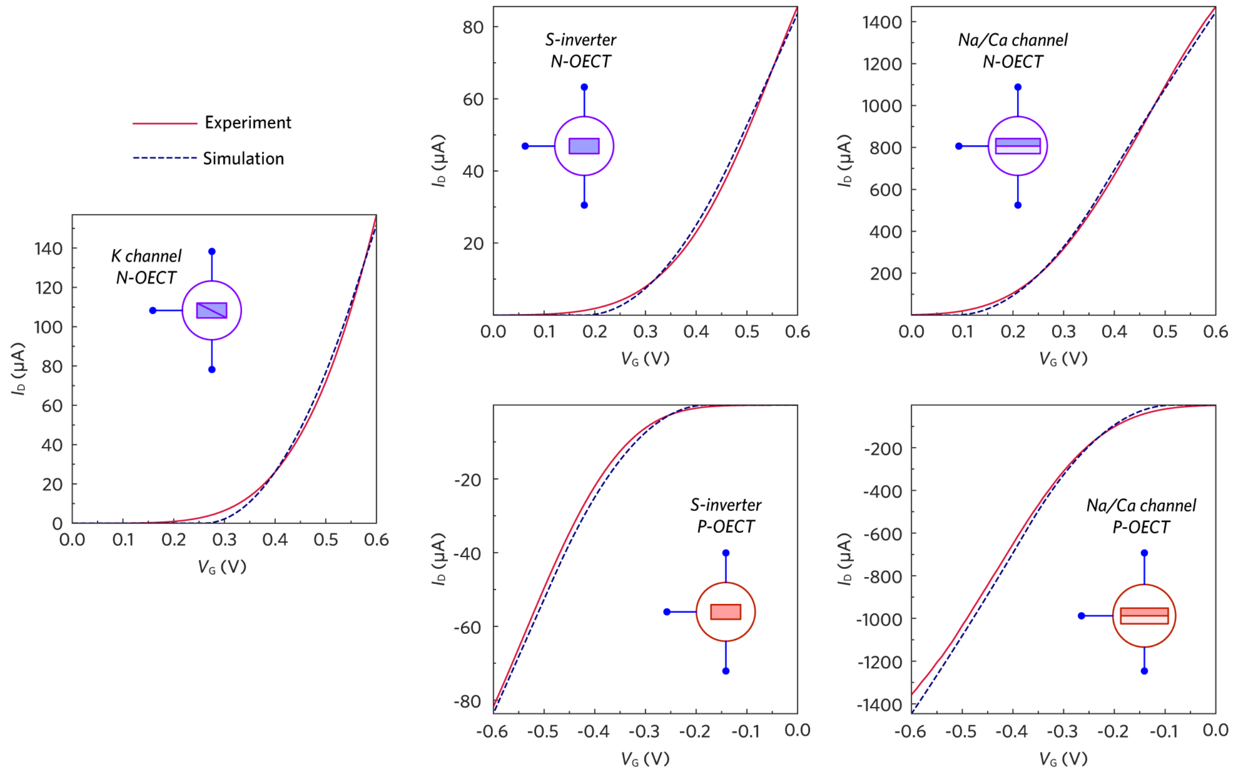

Supplementary Fig. 9 | Comparison of simulated and experimental transfer characteristics of all constituent OECTs in OECM.

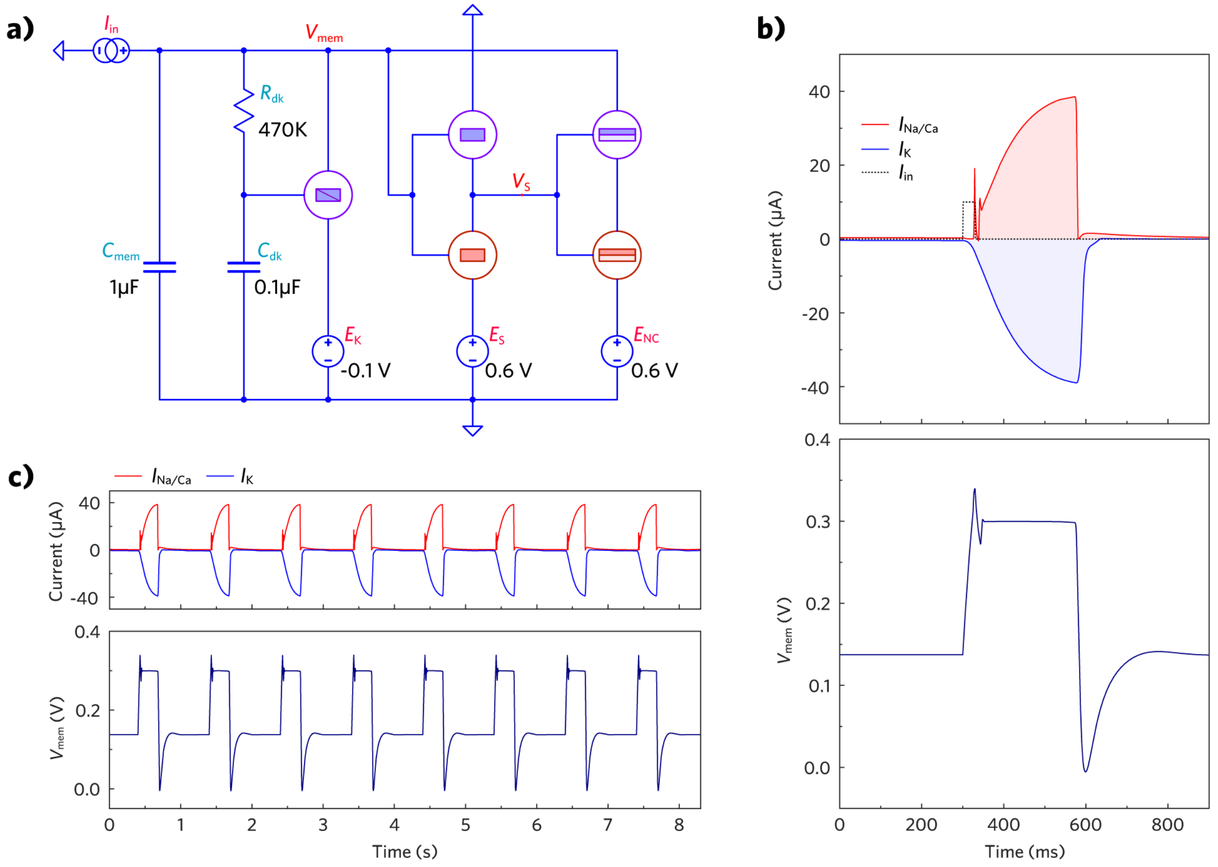

Supplementary Fig. 10 | SPICE simulation of OECM APs. (a) Equivalent circuit of OECM constructed in the SPICE software. **(b)** Simulative result of a typical OECM AP with charging/discharging currents. **(c)** Simulative result of a series of successive OECM APs.

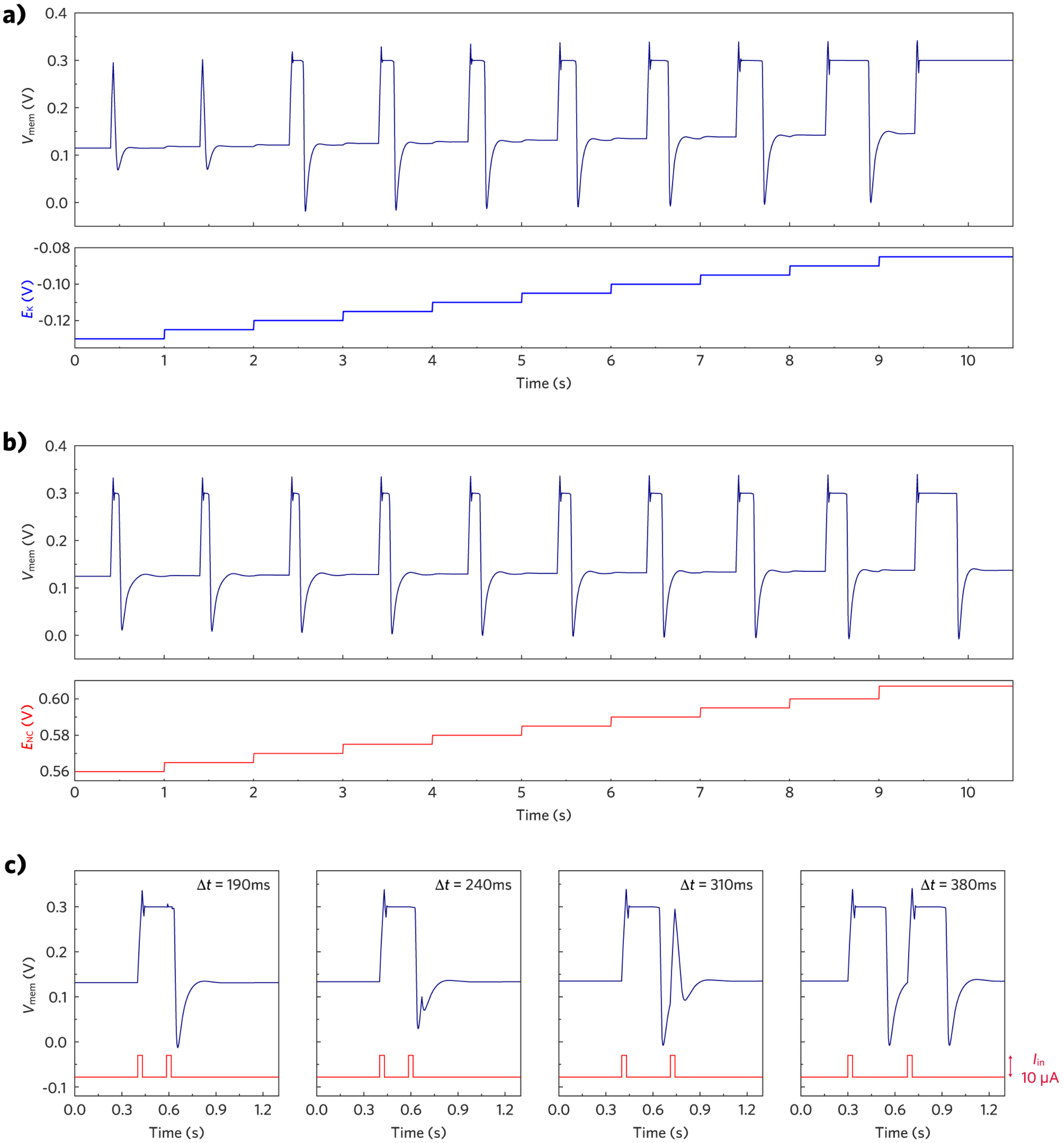

Supplementary Fig. 11 | SPICE simulation of OECM electrical modulation. (a) With E_K increasing from -130 to -90 mV, the simulated APD increased from 0 (no plateau) to ~510 ms. **(b)** With E_{NC} increasing from 560 to 605 mV, the simulated APD increased from ~120 to ~500 ms. **(c)** Simulated OECM refractoriness, exhibiting distinct absolute and relative refractory periods.

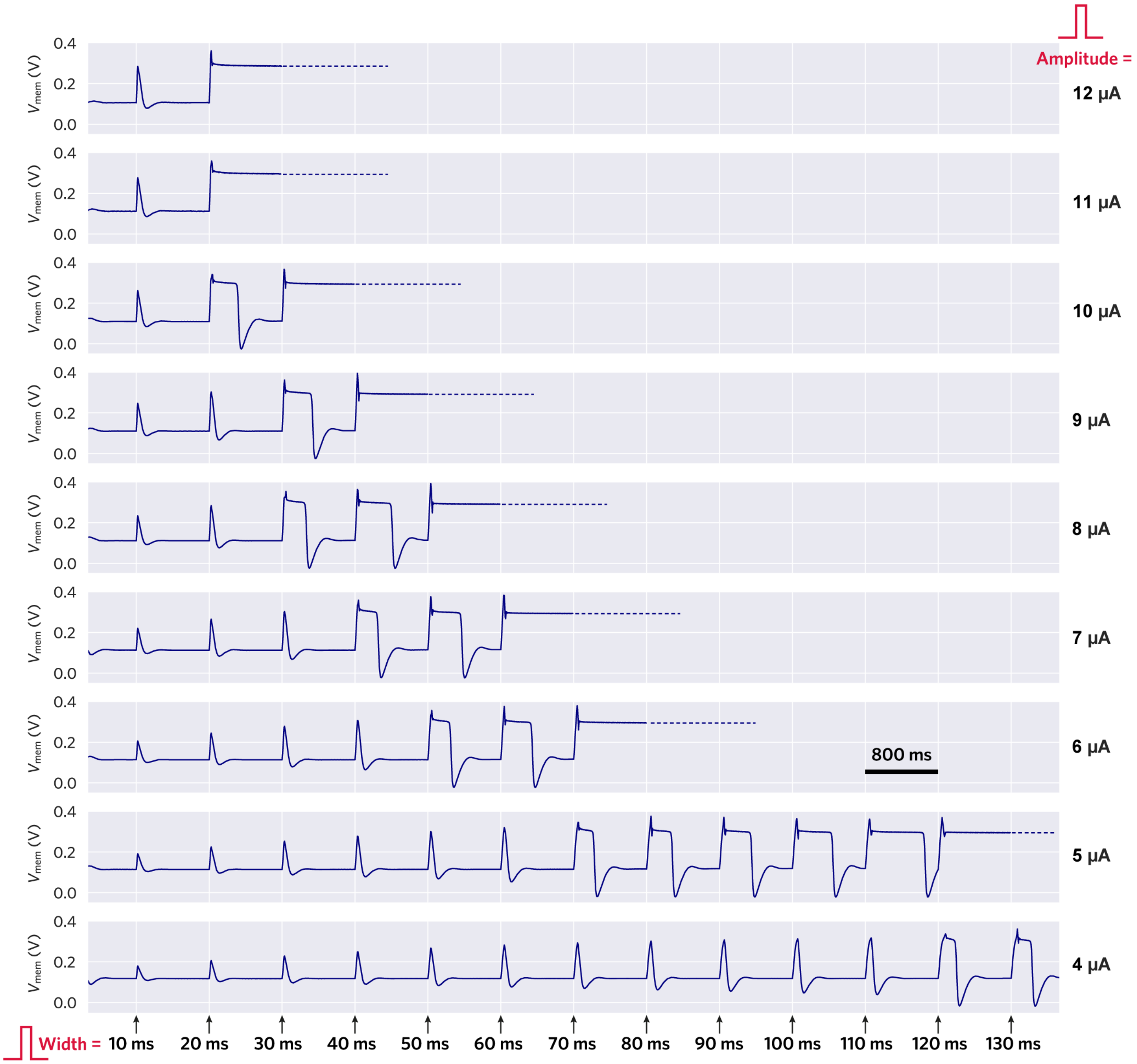

Supplementary Fig. 12 | The influence of I_{in} stimulus on OECM AP generation. Different I_{in} square pulses with varying width (10-130 ms) and amplitude (4-12 μA) were supplied to OECM as stimuli, resulting in different AP shapes. I_{in} amplitude determines the charging rate, while the multiplication of amplitude × width determines the total amount of charge supply. Inadequate charge supply leads to subthreshold spikes without cardiac plateau, whereas excessive charge supply prevents repolarization (dashed lines), similar to early afterdepolarization and reentry found in diseased vCMs.

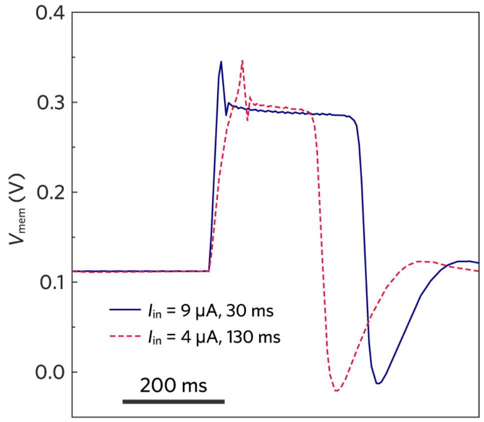

Supplementary Fig. 13 | The influence of I_{in} charging rate on OECM AP generation. I_{in} with larger amplitude produces higher upstroke velocity (9 μA: 4.7 V s^{-1}; 4 μA: 2.1 V s^{-1}), and demands a smaller pulse width to stimulate a full AP.

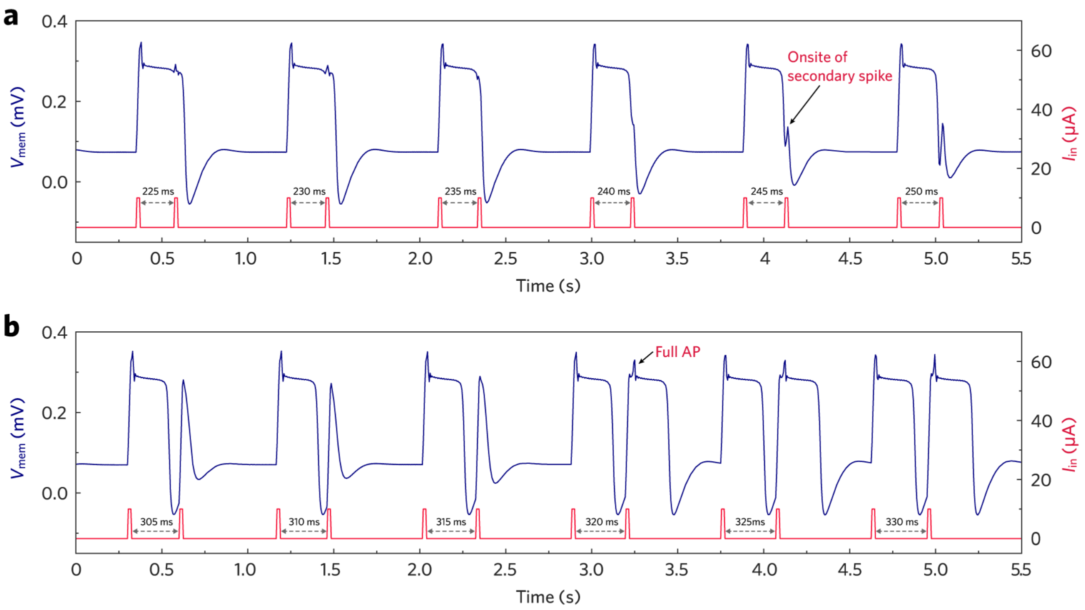

Supplementary Fig. 14 | Evaluation of OECM refractoriness periods. The duration of **(a)** absolute and **(b)** relative refractory period was evaluated by supplying afterdepolarization stimuli after the primary stimuli with increasing intervals (225 to 330 ms). The onsite of a secondary, ectopic spike suggests the transition from absolute to relative refractory period, while the generation of a full AP suggests the end of relative refractory period.

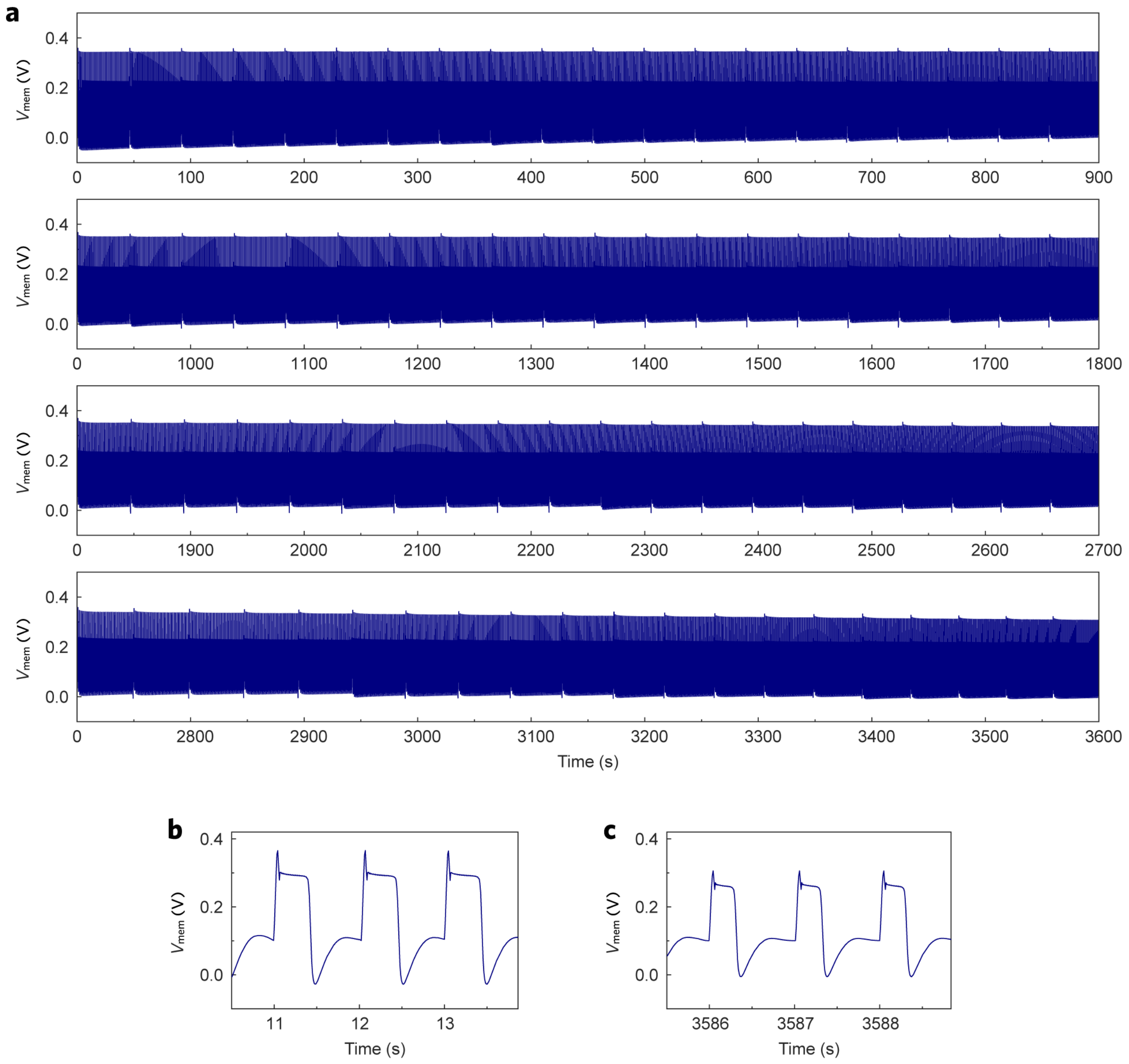

Supplementary Fig. 15 | Long-term stability of the OECM. (a) The OECM was continuously paced at 60 beats per minute for 1 h, producing 3600 APs in total. Due to instrument limitations in continuous recording duration, the measurement was performed as 90 consecutive runs of 40 s each, and the datasets were subsequently combined. The periodical "artifacts" correspond to the start of each new run. Zoom-in of OECM APs at the beginning **(b)** and end **(c)** of the cycling test. The variations in AP shape after prolonged operation primarily arise from the degradation of P($g_3$2T-TT), likely caused by oxygen-induced side reactions. The endurance of OECMs could be improved by suppressing oxygen reduction reactions (ORR)[7] or by employing more stable p-type organic mixed conductors, such as diketopyrrolopyrrole-based p-type materials[8].

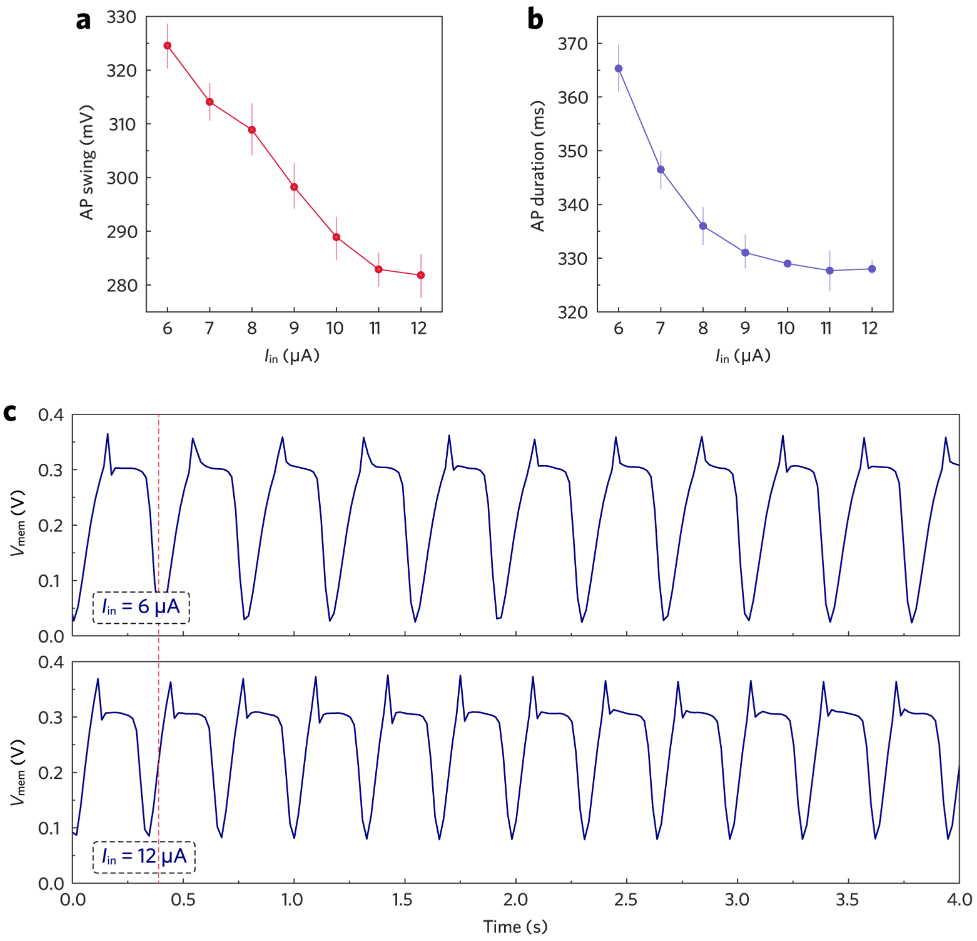

Supplementary Fig. 16 | Characterization of non-resting AP trains triggered by direct current injection. A higher stimulating current results in **(a)** reduced AP swing amplitude and **(b)** reduced AP duration. Error bar: 95% confidential interval, n = 10. **(c)** Comparison of OECM AP trains under different I_{in} level.

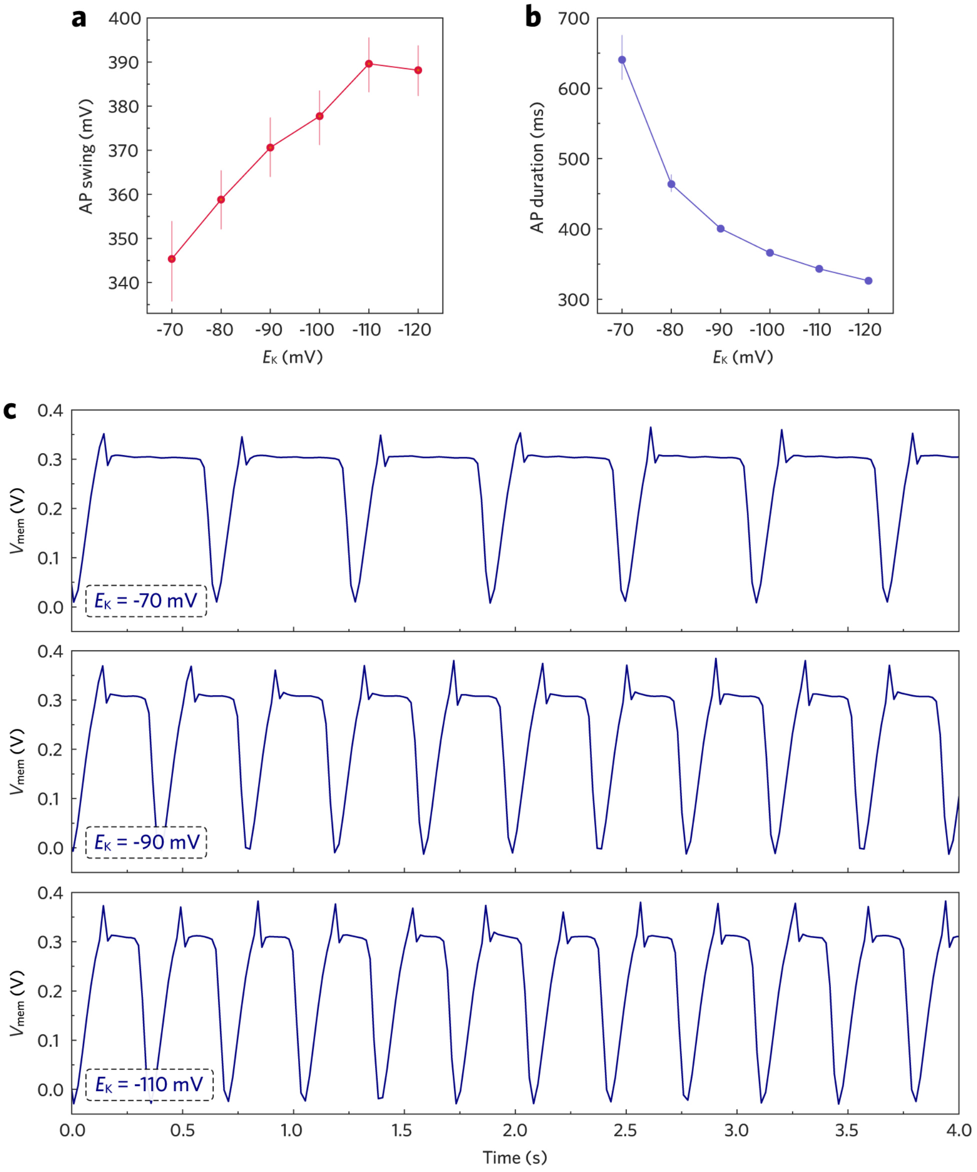

Supplementary Fig. 17 | Characterization of non-resting AP trains triggered by direct current injection. A more negative E_K results in **(a)** increased AP swing amplitude and **(b)** reduced AP duration. Error bar: 95% confidential interval, n = 10. **(c)** Comparison of OECM AP trains under different E_K bias.

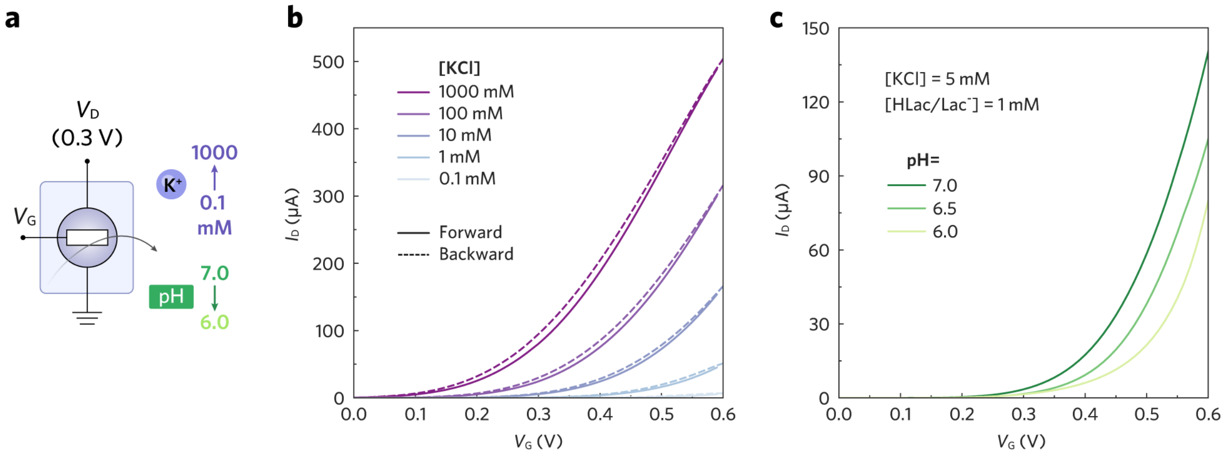

Supplementary Fig. 18 | Influence of electrolyte concentration and pH on the K channel's transfer characteristics. (a) Schematic circuit showing testing conditions. **(b)** Transfer curves of the BBL OECT measured under different [KCl] varying in logarithm scale. **(c)** Transfer curves of the BBL OECT measured under different pH values (i.e. proton concentrations). The electrolytes with pH = 6.0, 6.5, and 7.0 were prepared by firstly adding 1 mM lactic acid (HLac) into 5 mM KCl electrolyte, then titrating dropwise with a basic electrolyte (5 mM KCl, 1mM sodium bicarbonate ($NaHCO_3$)) while monitoring the pH value using a digital pH meter. The obtained electrolytes have a constant concentration in KCl (5 mM) and the conjugate HLac/Lac^- pairs (1 mM).

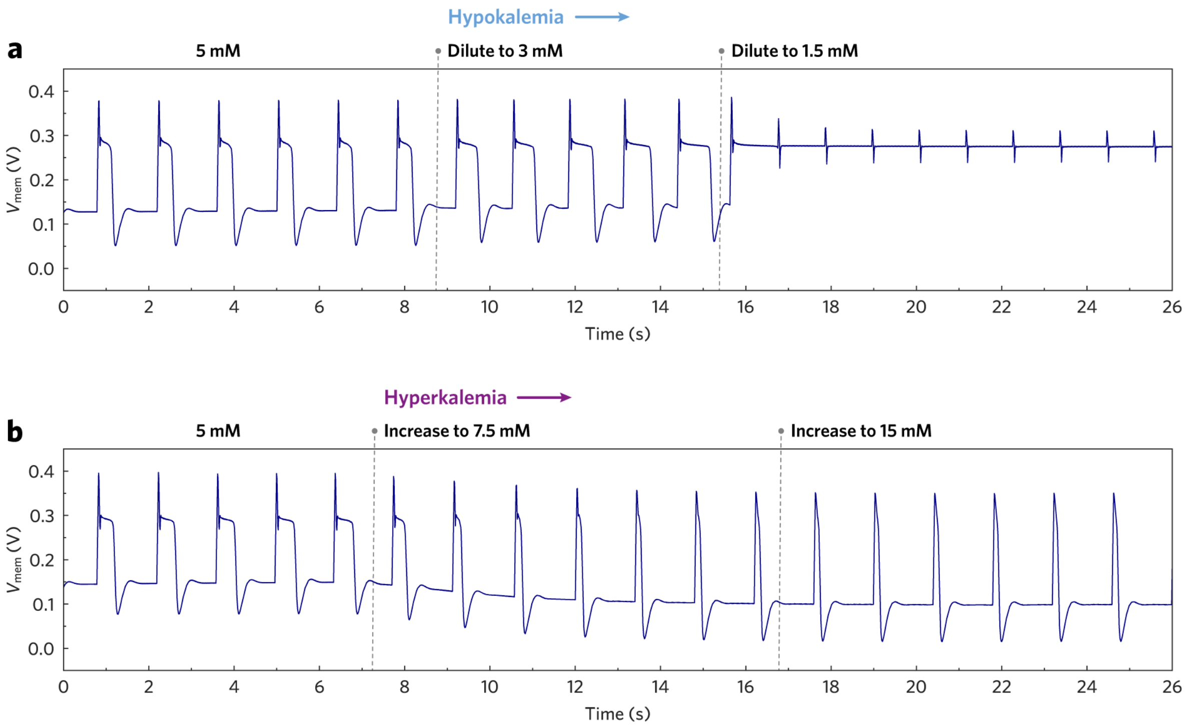

Supplementary Fig. 19 | Continuous recording of OECM AP trains while alternating ionic strength in the K channel's gating electrolyte. (a) To introduce hypokalemia, the OECT's gating electrolyte ([KCl] = 5 mM) was diluted by adding calculated amount of DI water. This resulted in prolonged plateau at [KCl] = 3 mM, and sustained depolarization at [KCl] = 1.5 mM. **(b)** To introduce hyperkalemia, calculated amount of high concentration electrolyte ([KCl] = 100 mM) was added into the OECT's gating electrolyte to elevate its ionic strength. This resulted in shortened plateau at [KCl] = 7.5 mM, and premature APs (missing plateau) at [KCl] = 15 mM.

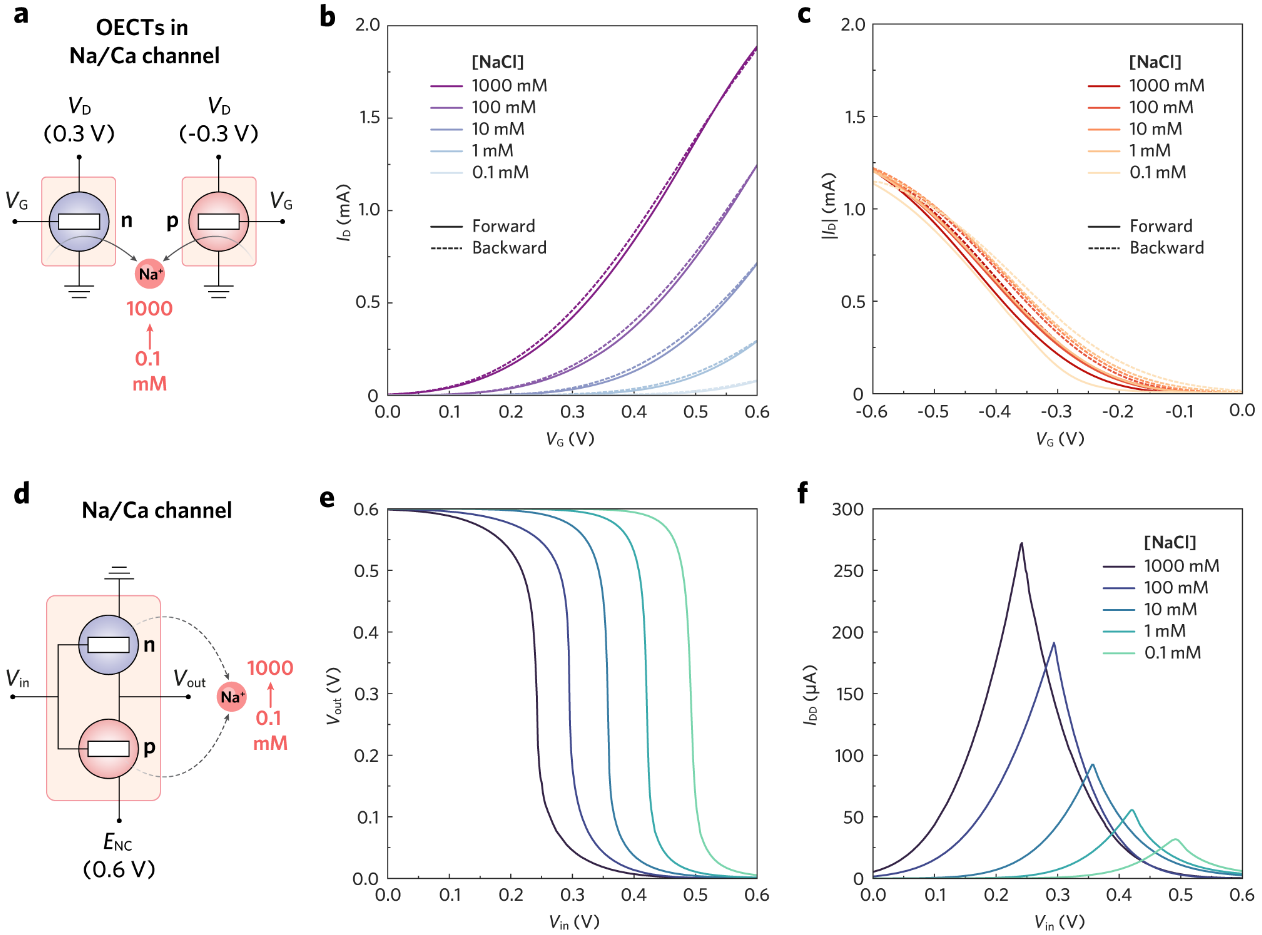

Supplementary Fig. 20 | Influence of electrolyte concentration on the Na/Ca channel's properties. (a-c) Transfer curves of the constituent OECTs were measured under different [NaCl] varying in logarithm scale. The n-type OECT's transfer behavior (on-off ratio, threshold voltage, etc.) correlates strongly with ionic strength. The p-type OECT shows little variation in its transfer behavior as [NaCl] varies from 1 mM to 1000 mM. Reducing [NaCl] to 0.1 mM slightly reduces the OECT's on-off ratio and threshold voltage. **(d-f)** VTC and CTC of the Na/Ca channel were measured under different [NaCl] varying in logarithm scale. The reduction in [NaCl] majorly impairs the n-type OECT's conductance, which shifts the inverter's transition voltage to a higher potential level and suppresses its supply current during switching.

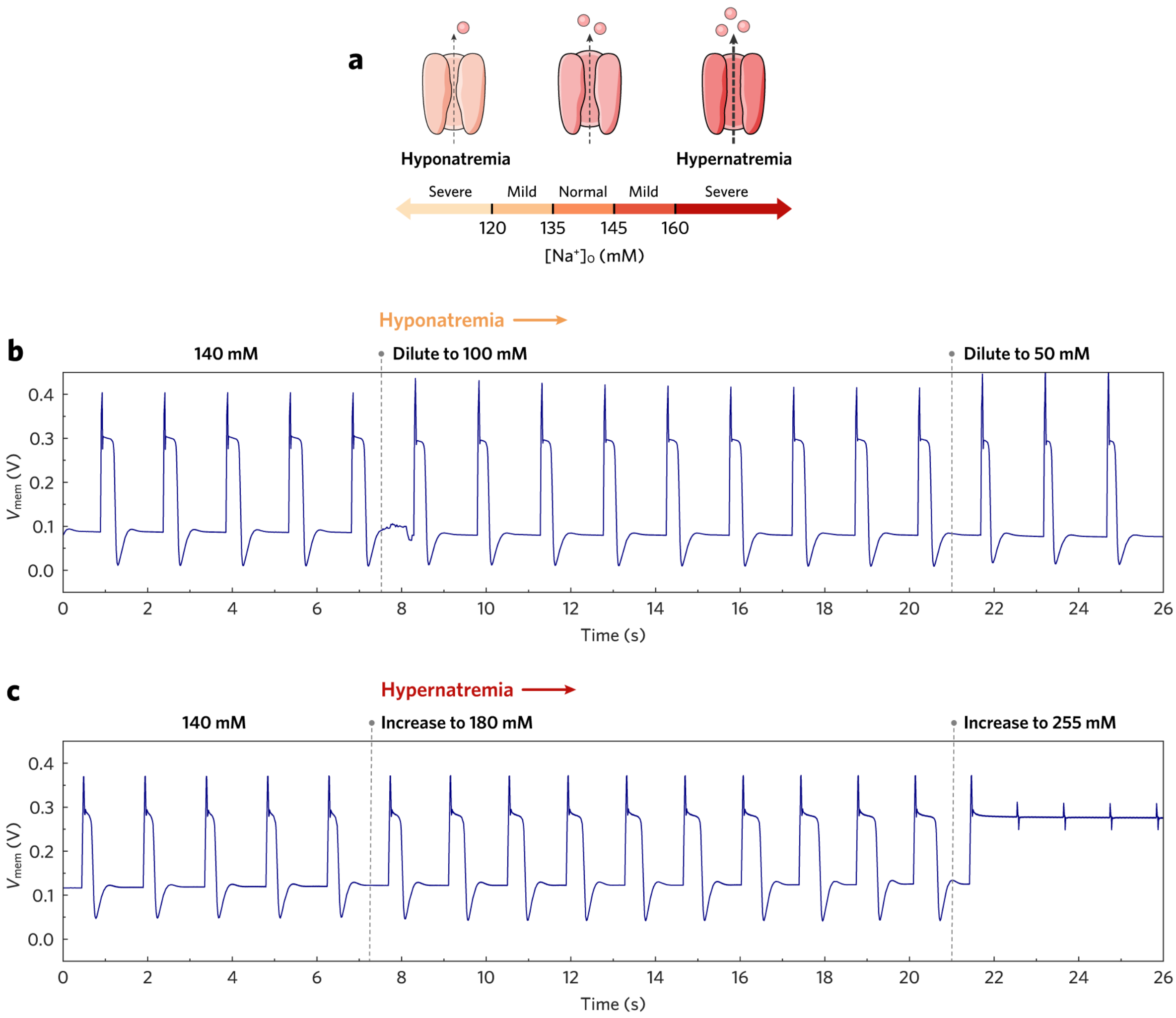

Supplementary Fig. 21 | Continuous recording of OECM AP trains while alternating ionic strength in the Na/Ca channel's gating electrolyte. (a) Schematic illustrations showing the concept of hypo- and hyper-natremia in vCMs. **(b)** To introduce hyponatremia, the OECTs' gating electrolyte ([NaCl] = 140 mM) was diluted by adding calculated amount of DI water. This resulted in shortened plateau at [NaCl] = 100 mM, and further shortened plateau at [NaCl] = 50 mM. **(c)** To introduce hypernatremia, calculated amount of high concentration electrolyte ([NaCl] = 1000 mM) was added into the OECTs' gating electrolyte to elevate its ionic strength. This resulted in prolonged plateau at [NaCl] = 180 mM, and sustained depolarization at [NaCl] = 255 mM.

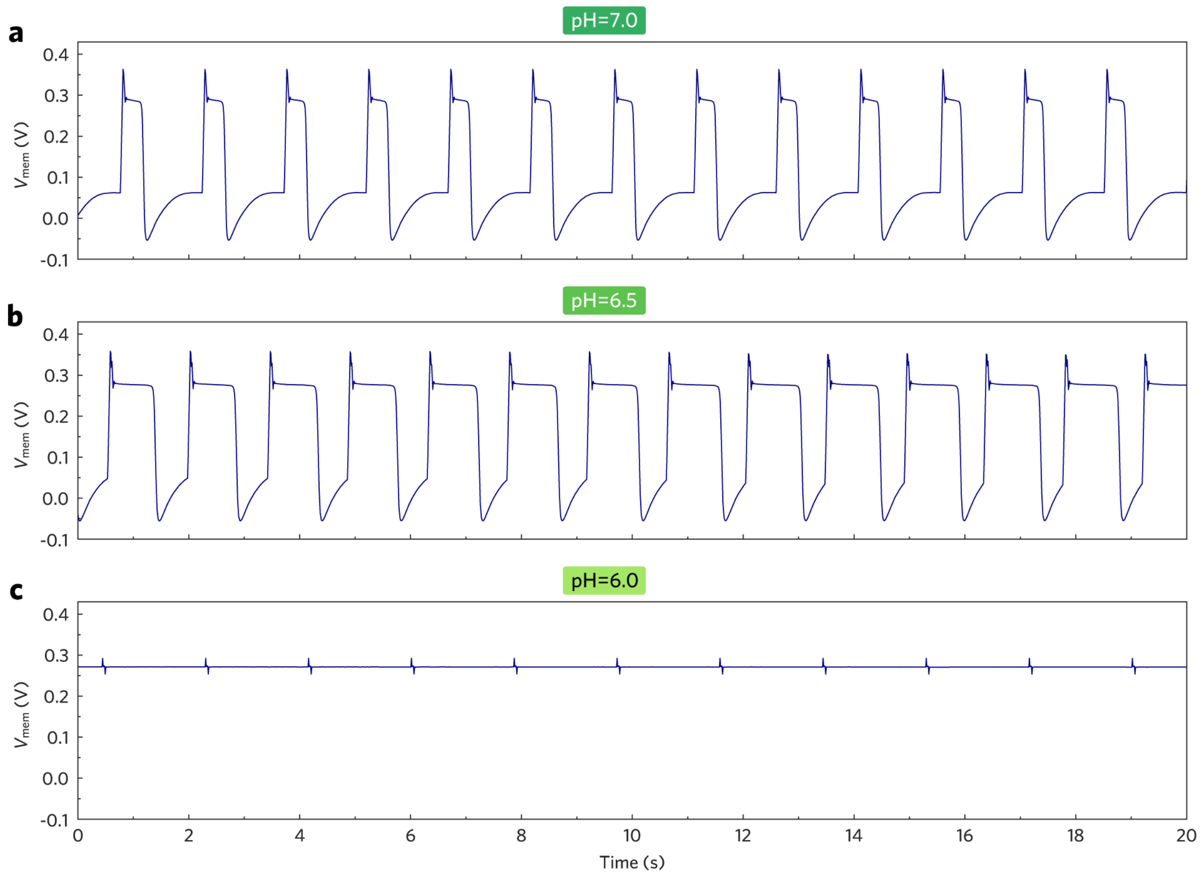

Supplementary Fig. 22 | Continuous recording of OECM AP trains with different pH in the K channel's gating electrolyte. (a) With a neutral electrolyte, the OECM could produce AP stably with an APD of ~380 ms. **(b)** With a slightly acidic electrolyte (pH = 6.5), the OECM's APD increased to ~900 ms. **(c)** With a weakly acidic electrolyte (pH = 6.0), the OECM could not repolarize with V_{mem} sustained at the depolarization level.

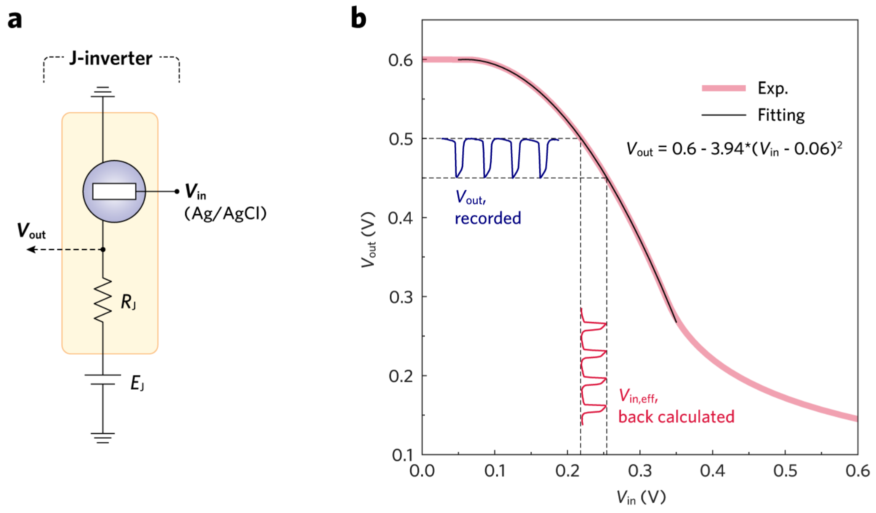

Supplementary Fig. 23 | Characterization and analysis of the junctional inverter (J-inverter). (a) Equivalent circuit of the J-inverter. R_J = 180 kΩ. **(b)** Standard VTC of the J-inverter measured using an Ag/AgCl pellet as gate, RPMI 1640 medium as electrolyte, without hiPSC-CMs between gate and channel. This curve correlates V_{out} and $V_{in,eff}$ because the Ag/AgCl gate is non-polarizable and thus the gate voltage can be entirely coupled onto the channel. The VTC was fitted into a quadratic function, through which $V_{in,eff}$ can be back calculated from V_{out} recorded during the *in vitro* experiment.

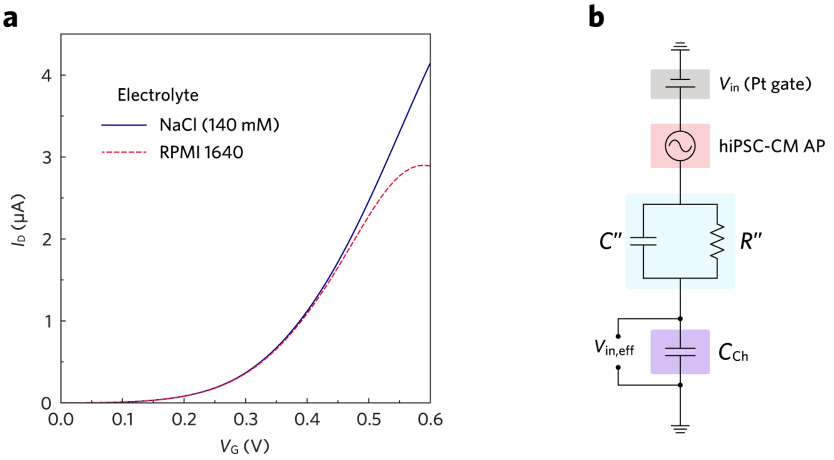

Supplementary Fig. 24 | Characterization and analysis of the biohybrid OECT. (a) A comparison of the OECT's transfer curves when using either 140 mM NaCl solution or the RPMI 1640 medium as electrolyte. Gate = Ag/AgCl, V_D = 0.3 V. **(b)** A simplified equivalent circuit describing the ionic circuit within the biohybrid BBL OECT. The circuit is composed of a direct voltage source (V_{in}) supplied by the polarizable Pt gate, an alternating voltage source (hiPSC-CM AP) that represents the AP pulses generated by hiPSC-CMs, damping elements (*R*" and *C*") that represent the collective impedance between the voltage sources and the BBL channel, and a capacitor (C_{Ch}) that represents the volumetric capacitance of BBL channel material. The effective input voltage to the J-inverter ($V_{in,eff}$) is defined as the potential drop coupled to C_{Ch}.

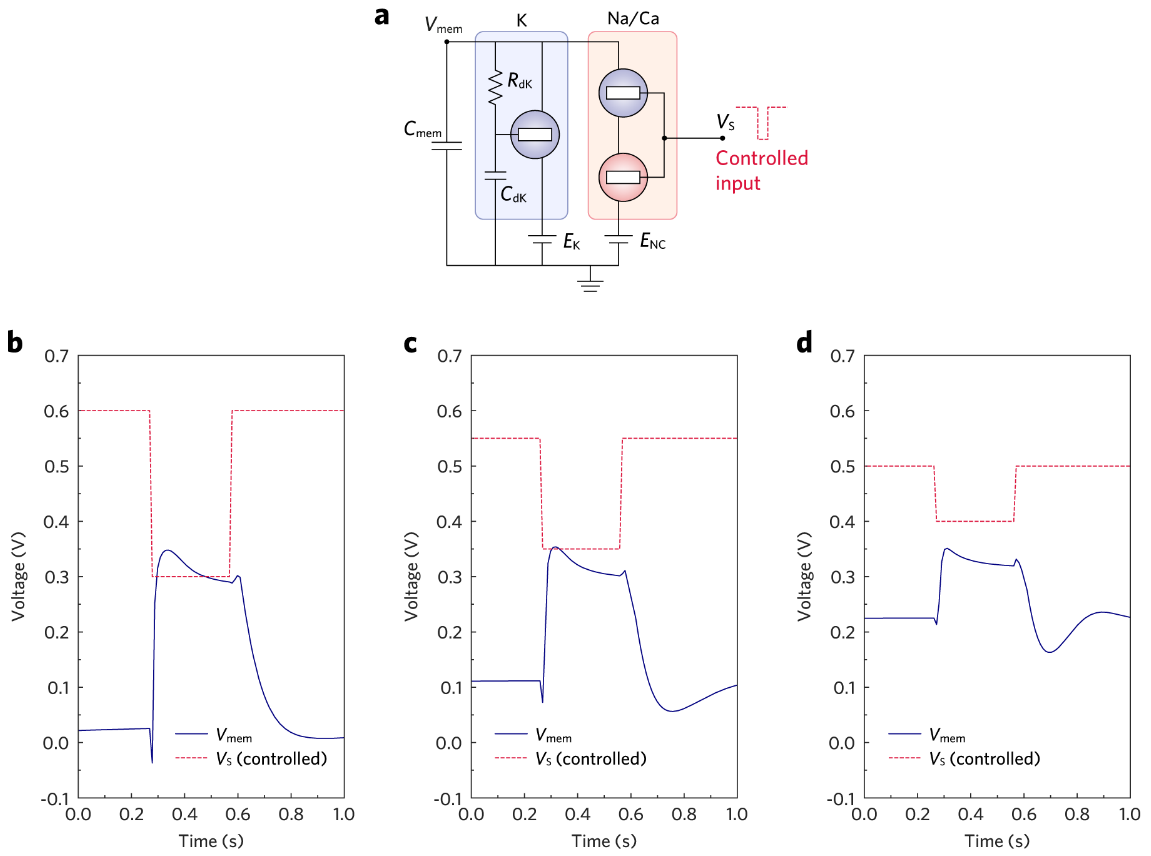

Supplementary Fig. 25 | Investigation on the OECM's AP shape with controlled V_S input. (a) Schematic circuit showing the testing condition. **(b-d)** Recorded V_{mem} output in response to stepped V_S input with varying amplitude. The full-range V_S step from 0.6 to 0.3 V produced a controlled AP similar to that generated by the self-regulated OECM. A reduction in the V_S step amplitude shifted the AP baseline upwards and reduced the AP swing amplitude, leading to an AP profile akin to that generated by the biologically coupled OECM. These results prove that the different amplitude in S- and J-inverter's output swing underpins the AP shape discrepancies between the self-regulated and biotic OECMs.

Supplementary Tables

Supplementary Table 1 | Ionic modulation of the OECM's AP characteristics by changing KCl concentration in the K channel OECT's gating electrolyte.

[KCl]	1.5 mM	3.0 mM	5.0 mM	7.5 mM	15 mM
Resting potential (%Chg.)	Sustained depolarization	165 mV (+6.5%)	155 mV (0%)	133 mV (-14.2%)	108 mV (-30.3%)
APD (%Chg.)	Sustained depolarization	865 ms (+101%)	430 ms (0%)	236 ms (-45.1%)	182 ms (-57.7%)

Supplementary Table 2 | Ionic modulation of the OECM's AP characteristics by changing NaCl concentration in the Na/Ca channel OECTs' gating electrolyte.

[NaCl]	50 mM	100 mM	140 mM	180 mM	255 mM
Resting potential (%Chg.)	78 mV (-15.2%)	82 mV (-10.9%)	92 mV (0%)	148 mV (+37.8%)	Sustained depolarization
APD (%Chg.)	300 ms (-28.9%)	356 ms (-15.6%)	422 ms (0%)	639 ms (+51.4%)	Sustained depolarization

Supplementary Notes

Supplementary Note 1 | The Beeler-Reuter ventricular model

In 1977, Beeler and Reuter developed the first ventricular action potential model (BR model)[1] by incorporating four of the eight distinct ionic currents postulated for ventricular cardiomyocytes (vCMs) at that time. This model was built upon the foundational framework of the Hodgkin-Huxley (HH) model, with an expansion to four currents and six gating variables. Specifically, the BR model implements a fast inward sodium current (i_{Na}), a slow inward calcium current (i_{s}), a delayed outward potassium current (i_{x1}), and an inward-rectifying potassium current (i_{K1}). Notably, i_{Na}, i_{s}, and i_{x1} exhibit both voltage and time dependence, whereas i_{K1} is only voltage-dependent. The rapid upstroke (phase 0) of the ventricular AP is primarily attributed to i_{Na}, while the subsequent currents play a major role in shaping the plateau (phase 2) and repolarization (phase 3) phases. The model implies that the change in a vCM's membrane voltage (V_{mem}) is the result of ionic current integration across its cell membrane capacitance (C_{mem}), which could be mathematically formulated by considering charge conservation:

$$C_{\mathrm{mem}} \frac{dV_{\mathrm{mem}}}{dt} = i_{\mathrm{Na}} + i_{\mathrm{s}} + i_{\mathrm{x}_1} + i_{\mathrm{K}_1} \tag{1}$$

where

$$i_{\mathrm{Na}} = (\overline{g_{\mathrm{Na}}}\boldsymbol{m}^3\boldsymbol{hj} + g_{\mathrm{NaC}})(E_{\mathrm{Na}} - V_{\mathrm{mem}}) \tag{2}$$

$$i_{\mathrm{s}} = \overline{g_{\mathrm{s}}}\boldsymbol{df}(E_{\mathrm{s}} - V_{\mathrm{mem}}) \tag{3}$$

$$i_{\mathrm{x}_1} = \overline{i_{\mathrm{x}_1}}\boldsymbol{x_1} \tag{4}$$

In the above equations, $\overline{g_{Na}}$ and $\overline{g_s}$ are the maximum conductance per unit area, while g_{NaC} is the constant portion of the conductance for i_{Na}. E_{Na} and E_{s} are the Nernst (reversal) potentials for sodium and calcium ions. $\boldsymbol{m}$, $\boldsymbol{h}$, $\boldsymbol{j}$, $\boldsymbol{d}$, $\boldsymbol{f}$, and $\boldsymbol{x_1}$ are voltage-time-dependent gating variables, each governed by a specific nonlinear differential equation. These variables model the probability of an ionic channel, or ratio of a cluster of ionic channels that is open under a specific V_{mem} and a given moment in time. $\overline{i_{x_1}}$ and i_{K_1} are formulated by a set of exponential components that take V_{mem} as the only variable. For the intricate mathematical descriptions of the BR model one can refer to the original publication by Beeler and Reuter[1].

As the first realistic model for ventricular action potential, the BR model unprecedentedly captured the overall waveform of the ventricular action potential. It elucidated the intricate relationship between voltage- and time-dependent conductance and the distinct phases of the ventricular AP, thereby establishing a foundational framework for subsequent cardiac models. Nonetheless, the BR model is not without its limitations in biological accuracy, particularly in its treatment of calcium and potassium currents, which stemmed from the gaps in knowledge regarding the various Ca^{2+} and K^{+} current types at the time of its development. Subsequent models, most notably the Luo-Rudy dynamic model, have enhanced the biological veracity by incorporating more accurately resolved ionic currents along with the inclusion of ion exchangers and pumps.

Despite the limitations, the BR model is still considered biophysically plausible and continues to find application in various research contexts such as computational cardiovascular physiology[2–5]. This work appreciates the BR

model for its mathematical simplicity and its adequate capacity to capture the essence of ventricular AP behavior. Hence, it is appropriately deemed an ideal conceptual framework for the construction of OECM.

Supplementary Note 2 | Mathematical descriptions of the OECM channels.

OECM functions by integrating the stimulative supply current (I_{in}, programmed square pulse) and the channel currents (I_{K} and I_{NC}) across the membrane capacitor (C_{mem}). In this architecture, E_{K} and E_{NC} act as fixed-potential batteries, biasing the channels in a manner akin to the Nernst potentials defined in the BR model. The conductance of the charging (Na/Ca) and discharging (K) channels is dictated by the device physics of organic electrochemical transistors (OECTs), whose behavior is dependent on both time and biasing conditions. The resulting fluctuations in V_{mem} is defined herein as the artificial cardiomorphic APs, which can be described via Kirchhoff's current law:

$$C_{\mathrm{mem}}\frac{dV_{\mathrm{mem}}}{dt} = I_{\mathrm{in}} + I_{\mathrm{NC}} + I_{\mathrm{K}} \tag{1}$$

Where I_{in} is a square pulse of current injection that serves as stimulation; I_{NC} and I_{K} are the electrical currents passing through the Na/Ca and K channels, respectively. By referring to the Bernards OECT model[6], the steady-state characteristics of K-OECT (in saturation region) can be described by

$$I_{\mathrm{K}} = -\mu_{\mathrm{K}} C_{\mathrm{K}}^{*}\frac{Wt}{2L}\left(V_{\mathrm{G}} - E_{\mathrm{K}} - V_{\mathrm{th,K}}\right)^2 \tag{2}$$

where μ_{K} and C_{K}^{*} are the charge carrier mobility and the volumetric capacitance density of the channel material (BBL); W, L, and t are the width, length, and thickness of the channel, respectively. V_{G} represents the effective gating voltage on the channel, which is equal to V_{mem} under steady-state conditions. E_{K} is the constant bias applied to the source terminal. $V_{\mathrm{th,K}}$ is the threshold voltage that is sensitive to the ionic strength in the OECT's gating electrolyte. At a KCl concentration of 5 mM, $V_{\mathrm{th,K}}$ was estimated to be ~0.29 V (Supplementary Fig. 7a).

The transient response of the K channel is governed by two distinct RC time constants existing at different levels of the circuitry. The first time constant arises from the electronic RC components that are directly coupled to the gate of the K-OECT, resulting in a delay characterized by $R_{\mathrm{dK}}C_{\mathrm{dK}} \approx 47$ ms. The second time constant, denoted as $\tau_{\mathrm{on,K}} = R_{\mathrm{i}}{*}C_{\mathrm{ch,K}}$, originates from the ionic circuit within the K-OECT. Here, R_{i} represents the ionic resistance of the electrolyte extending from the gate to the channel, while $C_{\mathrm{ch,K}}$ refers to the volumetric capacitance of the K-OECT's channel. R_{i} is inversely related to the concentration of the electrolyte. A lower ionic strength leads to an increased R_{i}, and consequently a prolonged $\tau_{\mathrm{on,K}}$. At a KCl concentration of 5 mM, $\tau_{\mathrm{on,K}}$ was experimentally estimated to be ~16.2 ms (Supplementary Fig. 2b). The transient response of $V_{\mathrm{G,trans}}$ is thus formulated as:

$$V_{\mathrm{G,trans}}(V_{\mathrm{mem}}, t) = V_{\mathrm{mem}}\left(1 - e^{-\frac{t}{R_{\mathrm{dK}}C_{\mathrm{dK}} + \tau_{\mathrm{on,K}}}}\right) \tag{2}$$

During OECM operation, the K-OECT operates in its saturation region given that ($V_{\mathrm{G,trans}}$ - $V_{\mathrm{th,K}}$) < V_{mem}. Combing equation (1) and (2), the voltage-time-dependent K-OECT characteristics is derived as

$$I_{\mathrm{K}}(V_{\mathrm{mem}}, t) = -\mu_{\mathrm{K}} C_{\mathrm{K}}^{*}\frac{Wt}{2L}\left[V_{\mathrm{mem}}\left(1 - e^{-\frac{t}{R_{\mathrm{dk}}C_{\mathrm{dk}} + \tau_{\mathrm{on,K}}}}\right) - E_{\mathrm{K}} - V_{\mathrm{th,K}}\right]^2 \tag{3}$$

The voltage-time-dependent current transfer characteristics (CTC) of the Na/Ca channel can be inferred from the behavior of its constituent OECTs. It is described by a set of functions that are contingent upon the relative potentials among $V_{\mathrm{S,trans}}$, E_{NC}, and V_{mem}:

$$I_{\mathrm{NC}} \approx 0, \quad V_{\mathrm{S,trans}} < V_{\mathrm{mem}} + V_{\mathrm{th,n}} \tag{4}$$

$$I_{\mathrm{NC}} = \kappa_{\mathrm{n}}\left(V_{\mathrm{S,trans}} - V_{\mathrm{mem}} - V_{\mathrm{th,n}}\right)^2, \quad V_{\mathrm{mem}} + V_{\mathrm{th,n}} < V_{\mathrm{S,trans}} < (V_{\mathrm{mem}} + E_{\mathrm{NC}})/2 \tag{5}$$

$$I_{\mathrm{NC}} = \kappa_{\mathrm{p}}\left(E_{\mathrm{NC}} - V_{\mathrm{S,trans}} + V_{\mathrm{th,p}}\right)^2, \quad (V_{\mathrm{mem}} + E_{\mathrm{NC}})/2 < V_{\mathrm{S,trans}} < E_{\mathrm{NC}} + V_{\mathrm{th,p}} \tag{6}$$

where $V_{\mathrm{th,x}}$ and κ_{x} ($x = n, p$) denote the threshold voltage and a set the material and geometrical properties of the complementary OECTs. $V_{\mathrm{th,x}}$ is a function depending on V_{mem}. κ_{x} is described as

$$\kappa_{\mathrm{x}} = \mu_{\mathrm{x}} C_{\mathrm{x}}^{*} \frac{W_{\mathrm{x}} t_{\mathrm{x}}}{2L_{\mathrm{x}}} \quad (x = n, p) \tag{7}$$

where μ_{x} and C_{x}^{*} are the charge carrier mobility and the volumetric capacitance density of the channel materials (BBL for n-OECT, $Pg_4$2T-TT for p-OECT); W_{x}, L_{x}, and t_{x} are the width, length, and thickness of the specific OECT channel, respectively.

When $V_{\mathrm{S,trans}} < V_{\mathrm{mem}}$, the Na/Ca channel is inactivated and supplies no charging current (Eq. 4). When $V_{\mathrm{S,trans}}$ is on the low-voltage side of the inverter's power rail, the n-OECT is biased in the saturation region, while the p-OECT is biased in the linear region (Eq. 5). When $V_{\mathrm{S,trans}}$ transits to the high-voltage side of the inverter's power rail, the n-OECT is biased in the linear region, while the p-OECT is biased in the saturation region (Eq. 6). The above equations are formulated by referring to the drain current of the OECT operating in saturation mode. In an ideal scenario where $\kappa_{\mathrm{n}} = |\kappa_{\mathrm{p}}|$ and $V_{\mathrm{th,n}} = |V_{\mathrm{th,p}}|$, the complementary inverter would exhibit a perfect symmetry in its voltage transfer characteristics (VTC) and CTC.

$V_{\mathrm{S,trans}}$ represents the effective voltage input coupling to the constituent OECTs' channels at a transient moment. It is described as

$$V_{S,trans}(V_{mem}, t) = V_S(V_{mem})\left(1 - e^{-\frac{t}{\tau_{on,S} + \tau_{on,NC}}}\right) \tag{8}$$

where V_{S} is the steady-state output of the S-inverter governed by its VTC; $\tau_{\mathrm{on,S}}$ and $\tau_{\mathrm{on,NC}}$ are the RC time constants originating from the ionic circuits in the S-inverter and the Na/Ca channel, respectively. The above equation implies that $V_{\mathrm{S,trans}}$ is a voltage-time-dependent function dictated by V_{mem} as well as two separable time constants. In contrast to the slow activation/deactivation rate in the K channel, the Na/Ca channel in OECM reacts to a $V_{\mathrm{S,trans}}$ change rapidly with a $\tau_{\mathrm{on,NC}}$ estimated to be ~0.17 ms (Supplementary Fig. 2a).

The aforementioned OECM channel currents mimic the ionic currents delineated in the BR model, with respective to their roles in the formation and regulation of membrane dynamics. Specifically, I_{K} plays an analogous role to i_{x1}, while I_{Na} and I_{Ca}, defined as I_{NC} during the forward and backward V_{mem} sweeps, correspond to the function of i_{Na} and i_{s}, respectively.

Supplementary Note 3 | Comparison of Organic Electrochemical Neurons and Cardiomyocytes

Leaky Integrate-and-Fire (LIF) OECN: This architecture is based on a compact Axon-Hillock (A-H) circuit, comprising an amplifying block (cascaded OECT-based inverters) and a resetting OECT (T_{reset}). The circuit operates by integrating an input current (I_{in}) onto a membrane capacitor (C_{mem}). When a threshold determined by the inverter's transition voltage is reached, a positive feedback loop triggers a rapid spike at V_{out}. This model is optimized for compact spiking logic and frequency modulation in neural networks, but is simplified with a lower level of biorealism. The inverters in a LIF OECN typically operate with fixed supply rails (High = V_{DD}, Low = GND). This provides high voltage gain and sharp switching, ideal for producing the rapid "all-or-none" spikes needed for neural information processing. Moreover, the cascaded inverters are not connected to V_{mem} and do not charge or discharge C_{mem}. T_{reset} serves the discharging function like the K channel in a biological neuron, but there is no explicit Na channel in a LIF OECN.

Conductance-based (HH) OECN: This circuit explicitly emulates the Hodgkin-Huxley (HH) framework. It utilizes two OECTs: a Na-OECT with ion-tunable antiambipolar behavior (mimicking sodium channel activation and inactivation) and a K-OECT with delayed activation. Both the Na-OECT and K-OECT are connected to $V_{mem,}$ allowing them to charge or discharge C_{mem} directly with dynamic supply rails. The Na-OECT is positioned between High = E_{Na} and Low = V_{mem}, while the K-OECT is positioned between High = V_{mem} and Low = E_K. The dynamic supply in HH OECNs mimics the biological driving force (V_{mem} - E_{ion}), where the strength of inward currents naturally diminishes as V_{mem} approaches the reversal potential. Such architecture mirrors the ion channels in biological neurons, allowing the HH OECN to capture the distinct conductances of individual ion channels and to emulate a wider range of neural features, such as latency, integration, and refractoriness.

OECM (this work): Designed according to the Beeler-Reuter (BR) model, the OECM moves beyond neural spiking to emulate the complex, multi-phase ventricular action potential (AP). It utilizes three functional blocks: an S-inverter, an inverter-based charging Na/Ca channel, and a delayed discharging K channel. Like the HH OECN, the OECM is also a conductance-based device, with its charging and discharging channels directly tied to V_{mem}, thereby creating dynamic supply rails. Specifically, the charging Na/Ca channel is positioned between High = E_{Na} and Low = V_{mem}, while the discharging K channel is positioned between High = V_{mem} and Low = E_K. The dynamic supply in OECM mimics the biological driving force (V_{mem} - E_{ion}), where the strength of inward currents naturally diminishes as V_{mem} approaches the reversal potential. The major difference between OECM and HH OECN is that OECM is uniquely configured to balance charging and discharging strengths is phase 2, which helps stabilize a long plateau that is crucial for excitation-contraction coupling. Besides, the OECM isolates the "sensing" function into a dedicated S-inverter block. By using a fixed E_S for this block, the OECM maintains a stable threshold for triggering the charging channel, even while the charging channel itself operates under dynamic power conditions.

In summary, the LIF OECN focuses on high-gain pulse generation, the HH OECN focuses on biorealistic neural features reproduction, while the OECM focuses on interdependent conductance balancing to shape a biorealistic, multi-phasic waveform. By adopting dynamic supplies, the OECM successfully shifts from a digital-like switching device to a biophysically grounded analog emulator of the human heart's electrical activity.

Supplementary Note 4 | Analysis of the OECM's primary and secondary equilibria

In the study of biological excitable cells, "equilibrium" typically refers to the resting potential (E_{rest}) — a stable fixed potential where the sum of all channel currents is zero. However, cardiac cells are unique in that their action potential (AP) exhibits a quasi-stationary state known as the plateau (phase 2). A **secondary equilibrium** occurs when a vCM reaches a temporary state of balance at a depolarized potential (away from E_{rest}). Unlike its **primary equilibrium** at the resting state, this is a dynamic metastable state in which inward and outward currents (e.g., SR calcium release flux and delayed rectifier potassium currents) are balanced. Because the conductances of these channels are time-dependent, this equilibrium is not permanent; it is a "moving target" that the system tracks until one current eventually dominates.

In the OECM, the secondary equilibrium arises from the interplay between the Na/Ca and K channels, mediated by the S-inverter. During phase 2, the S-inverter biases the Na/Ca channel into a high-conductance state, providing a sustained inward "charging" current that balances the outward discharging current from the K channel. The secondary equilibrium therefore corresponds to the depolarized voltage at which these opposing currents are equal, temporarily pausing the repolarization process. The balance is transient because of the intrinsic asymmetry in the channel dynamics. While the Na/Ca current is sustained by the S-inverter's bias, the K-channel exhibits slow ionic doping kinetics with a relatively long time constant. Consequently, during the plateau, the K-channel conductance is not static but continues to increase as more ions are injected into the BBL bulk. Once the outward current grows to exceed the inward current, the membrane potential begins to decline. This drop triggers a positive feedback loop: the decrease in V_{mem} is fed back through the S-inverter, shifting the Na/Ca-channel away from its conductance peak. This rapid "collapse" of the inward support current effectively terminates the secondary equilibrium and initiates the terminal phase 3 repolarization.

Supplementary References

1. Beeler, G. W. & Reuter, H. Reconstruction of the action potential of ventricular myocardial fibres. *J. Physiol.* **268**, 177–210 (1977).
2. Beeler-Reuter Mammalian Ventricular Model 1977 — Physiome Model Repository. https://models.physiomeproject.org/e/9a.
3. Cusimano, N., Bueno-Orovio, A., Turner, I. & Burrage, K. On the Order of the Fractional Laplacian in Determining the Spatio-Temporal Evolution of a Space-Fractional Model of Cardiac Electrophysiology. *PLOS ONE* **10**, e0143938 (2015).
4. Drovandi, C. C. *et al.* Sampling methods for exploring between-subject variability in cardiac electrophysiology experiments. *J. R. Soc. Interface* **13**, 20160214 (2016).
5. Halfar, R. Dynamical properties of Beeler–Reuter cardiac cell model with respect to stimulation parameters. *Int. J. Comput. Math.* **97**, 498–507 (2020).
6. Bernards, D. A. & Malliaras, G. G. Steady-State and Transient Behavior of Organic Electrochemical Transistors. *Adv. Funct. Mater.* **17**, 3538–3544 (2007).
7. Zhang, S. *et al.* Toward Stable p-Type Thiophene-Based Organic Electrochemical Transistors. *Adv. Funct. Mater.* **33**, 2302249 (2023).
8. Huang, W. *et al.* Vertical organic electrochemical transistors for complementary circuits. *Nature* **613**, 496–502 (2023).
